# Attitude of nurses and midwives towards collaborative care with physicians in Jimma University medical center, Jimma, South West Ethiopia

**DOI:** 10.1186/s12960-020-00531-6

**Published:** 2020-12-02

**Authors:** Eneyew Melkamu, Aynalem Yetwale

**Affiliations:** School of Nursing and Midwifery, Faculty of Health Sciences, Institute of Health, Jimma University, Jimma, Ethiopia

**Keywords:** Attitude, Nurses, Midwives, Physicians, Collaborative care

## Abstract

**Background:**

Good attitude on collaborative care between nurses and midwives with physicians is crucial for better team working. This further enables those vital health care professionals to provide quality and improved care for their clients.

**Objective:**

To assess the attitude of nurses and midwives towards collaborative care with physicians in Jimma University medical center, Jimma, South West Ethiopia.

**Methodology:**

The institution-based cross-sectional study was conducted from March 20 to April 8, 2019, using a semi-structured and standardized questionnaire. Study units were selected by simple random sampling using the lottery method. A total of 410 participants were included in the study. Data were entered into Epi data version 4.2 and exported to statistical packages for social sciences version 23 for cleaning and further analysis. Descriptive statistics were presented with tables, figures, and narratives. The level of significance was set at a *p* value of less than 0.05 in multivariable logistic regression.

**Results and conclusion:**

More than five out of ten, 234 (57.2%) of participants had a good (good attitude is defined in the operational definition section of methodology) attitude towards collaborative care with physicians, and the rest 175 (42.8%) poor attitude toward it. Participants had the highest median score in the shared education and teamwork (26.0) subscale and midwives were found to have higher mean rank scores compared to nurses. It was only in the nurse's/midwife's autonomy subscale that a statistically significant difference was found (*Z* = − 2.92, *p* value = 0.003). More generally, more than have of the participants had a good attitude on providing care collaboratively with physicians, though a significant proportion of nurses and midwives also rate collaborative care provision with physicians as poor.

**Recommendations:**

The findings of this study suggested that interventions are needed to be taken to improve nurse's and midwife’s attitudes on the provision of collaborative care with physicians. Enhancement of shared education, cooperation rather than dominance and caring attitude are all vital. These all could in turn enhance the quality of care provided for clients.

## Background

According to Carnwell and Buchanan (2004), collaborative care is defined as ''an intellectual and co-operative endeavor, knowledge and expertise more important than role or title, joint venture, team working, participation in planning and decision making, a non-hierarchical relationship, sharing of expertise, willingness to work together towards an agreed purpose, trust and respect in collaborators, highly connected network, and low expectation of reciprocation'' [1].

Health care provision needs many interactions and collaborations between different healthcare professionals with varying levels of education and professional qualifications [2]. Collaborative care benefits both care providers and consumers. Benefits for the care providers include: increased professional satisfaction due to clearer, more consistent goals of care and improved communication with other providers, enables the provider to learn new skills and approaches to care and work to full scope, provides an environment for innovation, and allows providers to focus on individual areas of expertise [3]. Benefits for the patient include: improves care by increasing the coordination of services, integrates health care for a wide range of health needs, empowers consumers as active partners in care, and results in better patient outcomes [3].

Though it was not possible to find studies assessing attitudes of nurses and midwives towards collaborative care with physicians at once (the same study assessing nurse’s and midwife’s attitude at the same time), studies conducted separately in different settings have shown that both nurses and midwives are short of good attitude [4–7]. A study conducted in Nepal indicated that only quarter of nurses (25.3%) had a good attitude towards collaborative care with physicians [4]. On the other side, in a study conducted in Saudi Arabia nurses were found to have better scores on the Jefferson scale of attitudes towards nurse–physician collaboration (JSATNPC) with a mean score of 51.2 ± SD 5.46 from the possible maximum score of 60 showing that nurses had a positive attitude towards working collaboratively with physicians[8].

Other studies undertaken in Germany and Malaysia had shown that nurses fail to have a positive or good attitude on their collaboration with physicians (more than half of nurses in both studies) said that they had a poor attitude on this aspect [9, 10]. The other study conducted in the North-Western part of Ethiopia also indicated that less than half (41%) of nurses had a poor attitude towards collaborating with physicians and had higher mean score on the shared education and teamwork subscale, 23.33 ± SD 3.07, and lowest score in the physician's dominance subscale, 5.87 ± SD 1.81[11].

Concerning midwives, it is difficult to find works of literature or documents which had assessed midwife's attitude towards collaborative care with physicians especially using the JSATNPC measuring scale. But, looking at a few works of the literature undertaken in different settings, midwives also face collaborative problems with physicians and found to have negative attitudes towards collaborative care with physicians. a systematic review which had assessed studies conducted in the United States (US), United Kingdom (UK) and Australia had shown that poor (negative) attitude towards collaborative care with physicians was a problem [12].

One more study done in the Netherlands showed that nurses and midwives rated their view towards collaboration with a physician as poor indicating that they both lack one core element for multidisciplinary quality health care, having a good attitude [13].

On top of the effort the government is taking to raise the number and quality of health services, it is vital to have a good attitude of nurses and midwives with the physician to provide collaborative care and thus enhancing the quality of care and making the work environment smooth and friendly. But, to the best of the author's knowledge, regarding the attitude of nurses and midwives on collaborative care with physicians, particularly those of midwives are not well explored and inadequate to notify health care providers and other concerned bodies in Ethiopia. Therefore, this study tried to fill these holes and examined the problem better.

## Methods

### Aims of the study

1. To assess the attitude of nurses and midwives on collaborative care with physicians at Jimma University medical center, Jimma, South West Ethiopia.
2. To explore the possible difference between nurses and midwives in attitude towards a collaborative care with physicians, at Jimma University medical center, Jimma, South West Ethiopia.

### Study area, period and population

This institution-based cross-sectional study was conducted from March 29 to April 12, 2019 G.C in Jimma University medical center which is located in Jimma town 352 km southwest of the capital of Ethiopia, Addis Ababa. Currently, it is the largest and the only teaching and referral hospital in the southwestern part of the country, providing services for approximately 15 million people in its catchment area.

It has 1600 staff of whom 648 are nurses and midwives (566 nurses and 82 midwives). The hospital also has 800 beds and provides many health care services in the gynecology and obstetrics, internal medicine, pediatrics, emergency, radiology, surgery, and other departments.

The study population was nurses and midwives with a length of service half a year and above (as they are considered to have more experience of working with physicians in addition to being considered full employees by the civil service law of Ethiopia) and those available during the study period.

### Sample size and sampling procedure

The sample size (*n*) was calculated using the formula to estimate a single population proportion:

*n* = [(Zα/2)2 p (1−p)/d2]. Then the minimum sample size: *n* = (1.96)2 (0.41) (0.59)/(0.05)2 = 371.71 ≈ 372 taking *p* = 0.41 from a previous study done in North West Ethiopia [11].

Adding 10% for non-response rate, the final sample size was calculated to be: *n* = 372 + 37.2 ≈410. Using population proportion formula: ni = Ni × n/N, number of nurses = 566 × 410/648 = 358.12≈358 and number of midwives = 82 × 410/648 = 51.88≈ 52. Therefore, a total of 358 nurses and 52 midwives were included in the study.

A stratified sampling technique was used to select the study population. The study population was stratified by profession to nurses and midwives and the sample was taken from each stratum proportionally. Individual participants were selected using simple random sampling with a lottery method to attain the final sample size. A list of nurses and midwives from each ward was used as a sampling frame.

### Operational definitions

#### Good attitude of nurses and midwives towards collaborative care with physicians

Higher factor score on the adapted Jefferson scale of attitude towards nurse–physician collaboration (55 and above).

#### Poor attitude of nurses and midwives towards collaborative care with physicians

Lower factor score on the adapted Jefferson scale of attitude towards nurse–physician collaboration (below 55).

### Data collection instrument and procedure

Data collection was facilitated by five trained data collectors who have a BSc degree. The sociodemographic characteristics were assessed using close-ended questions. The attitudes of nurses and midwives were assessed by the adapted version of the Jefferson Scale of Attitudes toward Nurse Physician Collaboration. The final version of the JSAPNC contains 15 items answered on a 4-point Likert-type scale from (1 ''strongly disagree'' to 4 ''strongly agree''). A higher total score reflects a more positive attitude toward physician–nurse collaborative relationships". The instrument has known factors that were identified as: * 'shared education and teamwork' (7 items), 'caring as opposed to curing' (3 items), 'nurse's autonomy' (3 items) and 'physician's authority'(2 items).

The tool was originally developed by Hojat and Herman in 1985 and was modified in 2003 by Hojat et al. [14]. This tool was supported by psychometric evidence including construct validity and internal consistency reliability that can be used as a research tool in western countries. According to Hussein SZ1, Fatin Amira Ahmad and S.Hawa M.Noh (2018), the tool has good internal consistency with a Cronbach’s alpha coefficient reported of 0.87. In the current study, it was found to have a Cronbach’s alpha coefficient of 0.72 which is within the acceptable range. To check whether it works in Ethiopia, a pretest was conducted by a study done in North West Ethiopia in Goba referral hospital and finally confirmed that the tool can be applied in the Ethiopian context [11]. This tool (JSAPNC) was preferred from other psychometrically supported instruments available for measuring nurse’s and midwife’s attitude on collaborative care with physicians as it was applied in the Ethiopian context and confirmed to be valid and reliable to be used in Ethiopian studies.

Data were collected by administering a written questionnaire to study participants which were prepared originally in English and then translated into Affann Oromo and Amharic (the local languages in the study area) by a language expert in all those three languages. Then, it was translated back to English to keep its consistency and validity.

### Data quality control

To maintain the quality of the data, a pretest was done on 41 (10%) of nurses and midwives in a different hospital found in Jimma city, South West Ethiopia, and necessary modifications including wordings were made on the questionnaire before it was applied on the study population. Furthermore, each questionnaire was checked for completeness before data entry. Data were interred in software called Epi data version 4.2 to point out errors made during data collection automatically then transferred to SPSS version 23. Furthermore, training was given to data collectors and supervisors. The overall data collection process was monitored by supervisors**.**

### Data processing and analysis

The collected data were entered into epi data version 4.2 and exported to SPSS version 23 for cleaning and further analysis. Again, a *p* value of less than 0.25 in binary and 0.05 in multivariable logistic regressions was considered as significant at a 95% confidence level. Mann–Whitney U test was used to evaluate the possible difference between nurses and midwives on attitude towards collaborative care with physicians. Results were presented by frequency tables, percentages, measures of central tendency, and dispersion and statements.

## Results

### Sociodemographic characteristics of study participants

A total of 409 participant data were considered for analysis as there was one incomplete questionnaire yielding a response rate of 99.76%. The mean age of the respondents was 32.14 (SD ± 7.12) years. The other sociodemographic characteristics of study participants are presented with Table 1.

**Table 1:** Sociodemographic characteristics of respondents (*n* = 409) in Jimma University medical center, Jimma, South West Ethiopia

Characteristics	Frequency	Percentage
Sex		
Male	234	57.2
Female	175	42.8
Age		
20–29	166	40.5
30–39	183	44.6
40–62	69	14.9
Marital status		
Married	252	61.6
Single	157	38.4
Occupational status		
Ordinary staff (with no managerial role)	393	96.1
Head staff	16	3.9
Length of service in years		
0.5–10.5	332	81.2
10.6–20.6	56	13.7
20.7–32.0	21	5.1
Educational status		
Diploma		
Nurse	81	22.7
Midwife	6	11.5
BSc degree		
Nurse	273	76.5
Midwife	46	88.5
MSc degree and above		
Nurse	3	0,8
Midwife	_	_

### The attitude of nurses and midwives towards collaborative care with physicians

**Fig. 1:**
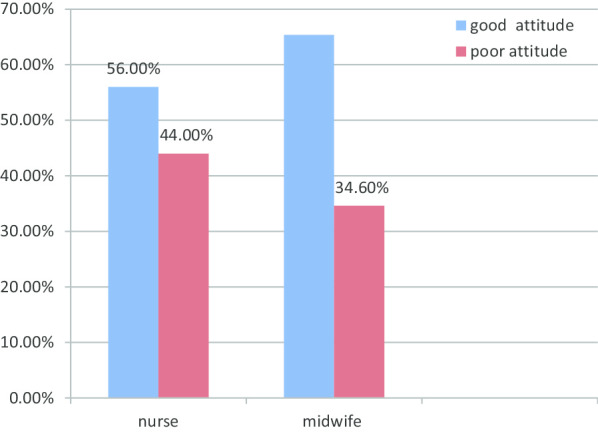
Classification of the attitude of nurses and midwives towards collaborative care with physicians at Jimma University medical center, Jimma South West Ethiopia. As shown in figure above, around two-thirds (65.4%) of midwives and more than half (56.0%) of the nurses had a good attitude towards collaborative care with physicians.

As indicated in Fig. 1 below, it seems more midwives had a good attitude (explained in the operational definition section of methodology ‘‘what is a good attitude towards collaborative care with physicians’’). Though, it may be due to the smaller sample size as compared to nurses as the number of nurses is more than six times that of midwives. The rest is shown in Fig. 1 and Table 2.

**Table 2:** Responses of participants on the attitude towards collaborative with physicians measuring items (*n* = 409) in Jimma University medical center, Jimma, South West Ethiopia

Items	Frequencies			
	Strongly agree	Agree	Disagree	Strongly disagree
Shared education and teamwork subscale				
A nurse/midwife should be viewed as a collaborator and colleague with a physician rather than his/her assistant	320 (78.2%)	77 (18.8%)	7 (1.7%)	5 (1.2%)
Education is needed for physicians to improve collaborative relationships with nurses/midwives	301 (73.6%)	92 (22.5%)	12 (2.9%)	4 (1.0%)
The educational programs should include areas on team learning and interprofessional collaboration between physicians and nurses/midwives	276 (67.5%)	127 (31.0%)	6 (1.5%)	_
Nurses/midwives are responsible for monitoring the progress of treatment the patient is taking	290 (70.9%)	107(26.2%)	9 (2.2%)	3 (0.7%)
Nursing/midwifery and medical students should be involved in teamwork during their education in order to understand their respective roles	265 (64.8%)	131 (32.0%)	13 (3.2%)	_
Nurses/midwives and physicians share many areas of responsibilities	310 (75.8%)	85 (20.8%)	9 (2.2%)	5 (1.2%)
Decisions on hospital discharge of patients should be contributed by both physicians and nurses/midwives when the need arises	278 (68.0%)	123 (30.1%)	7 (1.7%)	1 (0.2%)
Physician's authority subscale				
Nurses/midwives are primarily responsible to accomplish the physician’s orders	21 (5.1%)	23 (5.6%)	125 (30.6%)	240 (58.7%)
Doctors should be the primary deciders in all health care matters of the hospital	24 (5.9%)	20 (4.9%)	113 (27.6%)	252 (61.6%)
Nurse’s/midwife’s autonomy subscale				
Nurses/midwives should participate in decisions with the hospital support services that affects their work	275 (67.2%)	120 (29.3%)	10 (2.4%)	4 (1.0%)
Nurses/midwives should be accountable for their care to the patient	304 (74.3%)	89 (21.8%)	11 (2.7%)	5 (1.2%)
Nurses/midwives should clarify a physician’s order when they feel that it might have the potential for unfavorable effects on the patient	257 (62.8%)	121 (29.6%)	25 (6.1%)	6 (1.5%)
Caring as opposed to curing subscale				
Nurses/midwives should be involved in making policy decisions influencing their working conditions	279 (68.2%)	112 (27.4%)	12 (2.9%)	6 (1.5%)
Nurses/midwives have their own expertise in patient education and counseling	307 (75.0%)	98 (24.0%)	4 (1.0%)	_
Nurses/midwives are qualified to assess and solve psychological aspects of patient needs	328 (80.2%)	81 (19.8%)	_	_

### The response of nurses and midwives to the JSANPC (attitude towards collaborative care with physicians) items

In Table 3, it is illustrated that none of the participants neither disagree nor strongly disagree to the question “nurses/midwives are qualified to assess and solve psychological aspects of patient needs” and 253 (61.7%) of them strongly disagree with “doctors should be the primary deciders in all health care matters of the hospital”.

**Table 3:** Scores of participants on each subscale of the JSANPC in Jimma University medical center, Jimma, South West Ethiopia

Subscale	Median score	IQR	Minimum score	Maximum score
Shared education and teamwork	26	3	16	28
Caring as opposed to curing	12	1	7	12
Nurse’s/midwife’s autonomy	11	2	3	12
Physician’s dominances/authority	8	2	2	8

### The score of nurses and midwives on each subscale of JSANPC

As shown in Table 3, nurses and midwives had the highest median score in the ‘‘shared education and teamwork’’ and the lowest score was found in the ‘‘physician’s dominance/authority’’ subscale. This showed that nurses and midwives had no positive view of the physician’s dominance, but rather prefer to share education and care.

### Difference between nurses and midwives on their attitude towards collaborative care with physicians

The difference between nurses and midwives in the attitudes towards collaborative care with physicians was evaluated with the Mann–Whitney U test. This method is used to test for differences between two independent groups on a continuous measure. This test is the non-parametric alternative to the t-test for independent samples. Instead of comparing means of the two groups as in the case of the *t*-test, the Mann–Whitney *U* test actually compares medians. It converts the scores on the continuous variable to ranks across the two groups. It then evaluates whether the ranks for the two groups differ significantly. As the scores are converted to ranks, the actual distribution of the scores does not matter.

As shown in Table 4, midwives had a higher mean rank in all subscales compared to nurses and it is only in the nurse’s/midwife’s autonomy that a statistically significant difference was obtained.

**Table 4:** Difference in attitude towards collaborative care with physicians and separate scores of nurses and midwives in Jimma University medical center, Jimma, South West Ethiopia

JSANPC subscales	Profession	Median	IQR	Mean rank	Z-value	*p* value
Shared education and teamwork	Nurse	26.0	3.0	201.29	−1.69	0.09
	Midwife	27.0	3.0	230.50		
Caring as opposed to curing	Nurse	12.0	1.0	204.92	−0.04	0.97
	Midwife	12.0	2.0	205.54		
Nurse’s/midwife’s autonomy	Nurse	11.0	2.0	198.78	−2.92	*0.003 *
	Midwife	12.0	1.0	247.69		
Physician’s dominances/authority	Nurse	8.0	2.0	202.56	−1.19	0.23
	Midwife	8.0	2.0	221.73		

### Factors associated with the attitude of nurses and midwives towards collaborative care with physicians

As indicated in Table 5, nurses and midwives were not significantly different in all variables considered for the analysis based on the cross-tabulation result.

**Table 5:** Binary and multivariable logistic regression of variables associated with the attitude of nurses and midwives towards collaborative care with physicians at Jimma University medical center, Jimma South West Ethiopia

Variable	Frequency		COR, 95% CI	AOR, 95% CI	*p* value
	Good attitude	Poor attitude			
Age					
20–29	90	76	1	1	
30–39	100	82	1.030, 0.675–1.571	1.005,0.635–1.590	0.982
40–62	44	17	2.186, 1.155–4.135	2.174, 0.705–6.703	0.177
Marital status					
Single	86	71	0.851, 0.569–1.272	1.015, 0.651–1.583	0.947
Married	148	104	1	1	
Level of education					
Diploma	52	35	1.143, 0.706–1.850	1.241, 0.757–2.032	0.392
BSc degree and above	182	140	1	1	
Profession					
Nurse	200	157	0.674, 0.367–1.239	0.634, 0.340–1.182	0.152
Midwife	34	18	1	1	
Occupational status in the hospital					
Ordinary staff	221	169	0.297, 0.083–1.057	0.436, 0.115–1.660	0.224
Head staff	13	6	1	1	
Length of service in years					
0.5–10.5	181	151	0.738, 0.298–1.827	1.534, 0.395–5.954	0.536
10.6–20.6	40	16	1.538, 0.536–4.416	1.809, 0.579–5.652	0.308
20.7–32.0	13	8	1	1	

‘‘1’’ in Table 5 indicates category used as a reference in the analysis

“AOR”: adjusted odds ratio

‘’COR”: crude odds ratio

## Discussion

It is noted that more than half of nurses and midwives had a good attitude towards collaborative care with physicians. But, it could not be forgotten that more than one-third of them also rate their attitude towards collaboration with physicians as poor which is an important figure.

In this study, around five out of ten (56%) of nurses and nearest to two-thirds (65.4%) midwives had a good attitude towards collaborative care with physicians. Almost similar findings were obtained with studies conducted in Saudi Arabia, Tehran, and Palestine [7, 15, 16]. A better rating of good attitude was found in a study conducted in Nigeria (84%)[17]. This variation could have resulted from study time variation as today’s nurses and midwives are trained in a more harmonized teaching system that may help develop a good attitude to do in collaboration with physicians. Again the difference may come as a result of sample size variations and the point that in this study it is both nurses and midwives attitude towards collaborative care which is evaluated as a single population and this may have influenced the overall attitude of the participants.

Lower figures of attitude towards collaborative care with physicians were noted in studies conducted in Canada, the Netherlands and Ethiopia [11, 18, 19]. These variations may come from the difference in the study area, socio-cultural variations sample size, study period, and the way the data are collected and analyzed as those referenced studies assessed the attitude of nurses and midwives towards collaborative care with physicians separately which could affect the figure obtained. Again, a Nepalese study has also shown that only a quarter of nurses had a good attitude towards collaborative care with physicians (25.3%) and on the contrary three-fourths of them rate their attitude as poor [4]. These differences may arise from variation in sample size as the Nepal study had a lower sample size than this study. Besides, the inconsistency could be due to a difference in the study area (as in the referenced article participants were from different health facilities), study time and method of analysis used.

In the present study, participants had the highest median score in the shared education and teamwork subscale (26.0) and the lowest median score was recorded in the physician’s dominance/authority subscale (8.0). This finding is almost consistent with many of the literatures considered for this study [6, 7, 11, 16, 18, 20–22]. This consistency on the issue in almost all works of literatures is expected. It could be because that human mostly did not need dominance and wants to share things rather than having commanding and subordinate interactions. Thus, the same is true in this case that nurses and midwives prefer to share and work as a team which seems a positive view than being dominated by physicians. Moreover, it is known that it is in the current times that health education curriculums are focusing on multidisciplinary education and teamwork which thus may impact collaborative caretaking in the work environment later on.

Another important finding of this study is that nurses and midwives did not significantly differ in the three subscales of the JSANPC (shared education and teamwork, caring as opposed to curing and physician’s dominance/authority) except the nurse’s/midwife’s autonomy (*p* = 0.003). Nurse’s/midwife’s autonomy was also a factor (made a significant difference) in studies conducted in Saudi government hospitals [16]. On the contrary, nurse’s autonomy was not a significant factor in the studies done in Mansoura University Hospital, Egypt (p = 1.854) and NorthWest Ethiopia (*p* = 0.223) [11, 20]. This variation could have resulted from sample size variation, the difference in the study area and analytical method used as it was the independent samples T-test which was used in both the referenced studies. Again, the change in the direction and emphasis of professional autonomy and the presence of clear professional roles and independence could make nurses and midwives value more for their professional autonomy and independence.

## Strengths and limitation

It was based on primary data which could improve the validity of the results obtained. The other strength is that the questionnaire was standardized (adapted standardized questionnaire) which increases the reliability and validity of the instrument. The limitation of the study may be the study setting as the study was conducted in one area that may to some extent border its generalizability.

## Conclusion

It is noted in this study that a higher proportion of nurses and midwives had a good attitude towards collaborative care with physicians. But, it should not be undermined that a significant figure of nurses and midwives also rate their collaborative care attitude as poor. This is a signal that interventions are needed to enhance their attitude as it would finally affect the quality of care provided for the client. A different attitude improving training and the creation of a smooth working environment is recommended. Again, from the four subscales, it was only the nurse's/midwife's autonomy that appeared to significantly differ which is indicative that how much nurses and midwives value their professional autonomy (midwives seem to value their autonomy more as shown with larger mean rank score). There was no variable found to significantly affect a participant’s attitude towards collaborative care with physicians. The authors also recognize the need to have a good attitude of physicians towards collaborative working with these two health care providers to create a smooth working environment.

## Data Availability

The datasets used for this study could be deposited in publicly available repositories where appropriate and upon reasonable request. All relevant raw data supporting the findings of this study can also be freely available from the corresponding author through email “enemelkamu@gmail.com “or with other means for researchers wishing to use them for non-commercial purposes without breaching participant confidentiality. There will not be any apprehension about the ethical aspect as participant data were de-identified.

## Abbreviations

AOR: Adjusted odds ratio
BSc: Bachelor Science
COR: Crude odds ratio
IQR: Inter-quartile range
JSANPC: Jefferson Scale of Attitudes towards Nurse–Physician Collaboration
SPSS: Statistical Packages for Social Sciences
